# Network modeling of kinase inhibitor polypharmacology reveals pathways targeted in chemical screens

**DOI:** 10.1371/journal.pone.0185650

**Published:** 2017-10-12

**Authors:** Oana Ursu, Sara J. C. Gosline, Neil Beeharry, Lauren Fink, Vikram Bhattacharjee, Shao-shan Carol Huang, Yan Zhou, Tim Yen, Ernest Fraenkel

**Affiliations:** 1 Department of Electrical Engineering and Computer Science, Massachusetts Institute of Technology, Cambridge, Massachusetts, United States of America; 2 Department of Biological Engineering, Massachusetts Institute of Technology, Cambridge, Massachusetts, United States of America; 3 Fox Chase Cancer Center, Philadelphia, Pennsylvania, United States of America; 4 Cancer Biology Program, Fox Chase Cancer Center; Philadelphia, Pennsylvania, United States of America; 5 Plant Biology Laboratory, The Salk Institute for Biological Studies, La Jolla, California, United States of America; 6 Genomic Analysis Laboratory, The Salk Institute for Biological Studies, La Jolla, California, United States of America; 7 Howard Hughes Medical Institute, The Salk Institute for Biological Studies, La Jolla, California, United States of America; University of Pittsburgh School of Medicine, UNITED STATES

## Abstract

Small molecule screens are widely used to prioritize pharmaceutical development. However, determining the pathways targeted by these molecules is challenging, since the compounds are often promiscuous. We present a network strategy that takes into account the polypharmacology of small molecules in order to generate hypotheses for their broader mode of action. We report a screen for kinase inhibitors that increase the efficacy of gemcitabine, the first-line chemotherapy for pancreatic cancer. Eight kinase inhibitors emerge that are known to affect 201 kinases, of which only three kinases have been previously identified as modifiers of gemcitabine toxicity. In this work, we use the SAMNet algorithm to identify pathways linking these kinases and genetic modifiers of gemcitabine toxicity with transcriptional and epigenetic changes induced by gemcitabine that we measure using DNaseI-seq and RNA-seq. SAMNet uses a constrained optimization algorithm to connect genes from these complementary datasets through a small set of protein-protein and protein-DNA interactions. The resulting network recapitulates known pathways including DNA repair, cell proliferation and the epithelial-to-mesenchymal transition. We use the network to predict genes with important roles in the gemcitabine response, including six that have already been shown to modify gemcitabine efficacy in pancreatic cancer and ten novel candidates. Our work reveals the important role of polypharmacology in the activity of these chemosensitizing agents.

## Introduction

Small molecule screens are a powerful tool to identify compounds that modify disease progression either directly or by synergistic action with existing drugs [1], [2]. However, identifying the pathways targeted by these molecules has been difficult, as often small molecules affect more than a single gene or pathway at once [3]. Here, we report a screen identifying kinase inhibitors that improve the efficacy of gemcitabine in pancreatic cancer. As is typically the case in such screens, although the compounds are often reported as each having one or at most a few target kinases, their actual effects are much broader. To make sense of these data, we developed a network-based approach that takes advantage of this promiscuity to identify targeted pathways.

Pancreatic cancer is one of the most aggressive cancers, with only 3% of patients surviving more than five years [4]. To date, the most commonly used chemotherapeutic agent in pancreatic cancer treatment is gemcitabine, a nucleoside analogue, which infiltrates the cell’s nucleotide metabolism, ultimately causing DNA damage and apoptosis [5]. In addition to causing DNA damage, gemcitabine exerts its cytotoxicity by inhibiting ribonucleotide reductase, the enzyme responsible for building deoxyribonucleotides from ribonucleotides [5]. However, despite its wide use, gemcitabine shows limited efficacy: only 20%-30% of cases show a response, and this response consists of only a minor increase in survival time and symptom alleviation after exposure to gemcitabine [4]. Given the urgent need for improved therapies, there has been considerable interest in identifying drugs that could function to improve the efficacy of gemcitabine.

Here we describe an integrative approach to better understand gemcitabine cytotoxicity. We performed a screen to identify kinase inhibitors that increase gemcitabine cytotoxicity in the pancreatic cancer cell line PANC1. To dissect the mechanisms by which these kinase inhibitors modulate the effects of gemcitabine, we used a network approach that integrates the screening data with additional epigenetic and transcriptional profiling data that we collected, and with published genetic data. We uncovered a network of pathways involved in the gemcitabine response in pancreatic cancer, and queried the network to propose novel candidate genes and pathways with predicted roles in increasing gemcitabine cytotoxicity. This approach allowed us to infer pathways supported by many of the targets of the kinase inhibitors of interest, leading to hypotheses about how the polypharmacology of kinase inhibitors, rather than the individual kinases they target, contributes to the cellular response to gemcitabine.

## Materials and methods

### Data and code

The sequencing and peak calling data used in this work can be found at GEO accession number GSE70810.

The code associated with this paper is at: http://github.com/oursu/Gem_code.

### Cell lines and growth conditions

We used PANC1 cells from American Type Culture Collection (ATCC), banked at Fox Chase Cancer Center (FCCC) until use. We cultured them in DMEM/10% FBS supplemented with 2 mM glutamine and 1% penicillin, streptomycin, and kanamycin (PSK) and maintained them at 37C in 5% CO2. When appropriate, we used charcoal stripped (FCCC cell culture facility) and dialyzed FBS (Life Technologies; 26400–036).

### A screen identifying kinase inhibitors that increase gemcitabine cytotoxicity

We seeded PANC1 cells into 384 well plates and treated with either vehicle (0.1% DMSO final concentration, used as a control) or with gemcitabine (20nM, a concentration shown to cause cells to arrest in S phase, but not cause cell death) [6]. After 24h, we added kinase inhibitors from a library consisting of 160 kinase inhibitors (Inhibitor Library I 384 well plate EMD Millipore) via pin tool to vehicle or gemcitabine-treated cells. We pre-diluted the compounds in DMSO to obtain indicated final concentrations. We determined cell proliferation 48h later using CellTiter-Glo^®^ (Promega). We detected luminescence from the cells using an EnVision plate reader (Perkin Elmer). We performed the experiments in duplicate.

Next, we determined how much each kinase inhibitor reduced cell viability of vehicle versus gemcitabine-pretreated cells at 8 concentrations of kinase inhibitor. We determined cell viability using the luminescence units (LU) from the plate reader, where control cells (DMSO-treated) were set to 100% viability. For each kinase inhibitor, we fit two Weibull distributions to model the cell viability as a function of kinase inhibitor concentration: one for the effect of the kinase inhibitor on vehicle treatment, and one for gemcitabine treatment. We fit the Weibull distributions using the R function “drm” (with the desired Weibull distribution selected as “W1.4”) from the R package “drc”. We excluded kinase inhibitors that could not be fit. We also excluded kinase inhibitors that were toxic in the vehicle treatment (viability below 50000 LU) at any concentration, since we were specifically looking for modulators of gemcitabine cytotoxicity without an effect in the control. We also excluded data points where the measurements for the vehicle treatment and those for gemcitabine treatment overlapped, since that reduced our confidence that viability is different in control versus gemcitabine treated cells. For the remaining compounds, we considered a kinase inhibitor changed gemcitabine-induced cytotoxicity, if the reduction in viability was > 30% compared to the vehicle treatment at that concentration. These data are available in S1 Table.

### Determining the kinases affected by each inhibitor (“kinase hits”)

We collected a dataset measuring in vitro effects of kinase inhibitors on kinase activity from [7]. Then, based on this dataset, we defined our set of kinase hits as those kinases whose activity is changed by more than 50% by a kinase inhibitor found to modify gemcitabine cytotoxicity. These data are available in S2 Table.

### Determining the genetic modifiers of gemcitabine efficacy (“genetic hits”)

To identify the genetic modifiers of gemcitabine efficacy, we analyzed data from a published genome-wide siRNA screen that measured how gemcitabine cytotoxicity was affected by the knockdown of each gene [8]. The screen quantified drug toxicity as cell viability reduction upon gemcitabine treatment compared to vehicle treatment. This reduction in cell viability was quantified as a Sensitivity Index (SI) computed in [8]. These data are summarized in S4 Table.

The following criteria define our set of genetic hits: i) FDR < 0.05, ii) SI deviation from 1 greater than 0.3, where genes with an SI > 1.3 enhance gemcitabine cytotoxicity and genes with an SI < 0.7 suppress gemcitabine cytotoxicity, and iii) survival of the knockdown is at least 50% compared to that of negative control upon vehicle treatment, to ensure candidate genes are not essential genes.

### RNA-seq experiments to measure gene expression changes induced by treatment with gemcitabine

To identify genes whose expression changes in response to gemcitabine, we performed RNA-seq on drug- and vehicle treated cells.

Briefly, we grew PANC1 cells for 24 hours and then treated them with either gemcitabine (at 50 nM), or vehicle (0.1% DMSO) for 48 hours. We then extracted RNA using the Qiagen Rneasy Plus Mini Kit and prepared sequencing libraries using the Illumina mRNA sample-preparation guide (cat# RS-930-1001). We sequenced the libraries via single end runs on an Illumina HiSeq Sequencer. We performed 2 biological replicates per condition.

We aligned reads to the human reference genome (hg19 assembly) using Tophat2 (version 2.0.8) [9]. From the aligned reads, we quantified transcript abundance as FPKM, using Cuffdiff (version 2.0.2) [10] with the Gencode V15 gene annotations (protein-coding annotations only), taking into account only genes with at least 10 reads (c parameter) [11]. This analysis is in S6 Table. The GO enrichment analysis, identifying pathways enriched or depleted in the genes differentially expressed upon gemcitabine treatment, is shown in S7 Table.

The following criteria define our set of differentially expressed genes in response to gemcitabine: i) q-value < 0.05, ii) fold change higher than 1.5, iii) minimum gene expression level of 0.1 FPKM in at least one of the conditions (consistent with previous observations of undetectable activity below similar thresholds [12], and iv) difference in FPKM between the two conditions of at least 1 FPKM, to filter out genes with artificially high fold-changes.

### DNaseI-seq to measure changes in chromatin accessibility induced by treatment with gemcitabine

To identify changes in gene regulation induced by gemcitabine, we profiled changes in chromatin accessibility by DNaseI-seq on drug- and vehicle treated cells.

Briefly, we digested isolated nuclei with DNaseI for 1 minute (40 units DNaseI per 5 million cells) and then we followed the protocol described in [13]. We sequenced the libraries as single end runs on a HiSeq machine. We used one biological replicate per condition.

To identify accessible chromatin regions, we used the MACS algorithm [14], to identify peaks of DNaseI hypersensitivity genome-wide. As in the RNA-seq analysis, we used the hg19 genome assembly. For each condition, we first identified high-quality peaks with MACS, using a p-value cutoff of 1e-5. For all downstream analyses, to increase our sensitivity for detecting open chromatin regions, we considered a master set of peaks composed of: i) peaks from gemcitabine treated and vehicle treated samples, and ii) peaks from the DNaseI hypersensitivity dataset available on ENCODE for the PANC1 cell line [15].

### Running the SAMNet algorithm

To integrate the distinct biological datasets into a network model we used SAMNet, a multi-commodity flow-based network approach that links sets of genes of interest (in our case, the genetic hits, the kinase hits, the differentially expressed genes) through a known protein-protein interaction network and a derived TF-gene network, in a constrained optimization setting [16]. Code for SAMNet can be found at http://www.github.com/sgosline/SAMNet.

We considered two simultaneous analyses (referred to as “commodities” in SAMNet formulation) linked to the observed transcriptional response: first, pathways related to genetic hits and second, pathways related to kinase hits. For each commodity, SAMNet takes as inputs the respective genetic/kinase hits, and tries to connect those hits to the set of observed differentially expressed genes, through protein-protein interactions or protein-DNA interactions, thus pointing to novel genes likely involved in the biological process that have escaped experimental detection. The algorithm involves sending an imaginary flow the kinase hits and genetic hits, through the network to the differentially expressed genes. All network edges have a capacity limiting the amount of flow that can be sent through them. The optimization attempts to maximize the number of input genes included in the network (more precisely, it maximizes the total flow sent through the network), while minimizing the number of edges (protein-protein and protein-DNA interactions) it uses to make these connections. To account for the variable confidence associated with each protein-protein and protein-DNA interaction, the cost paid for each edge is inversely proportional to the edge’s confidence. Similarly, we prioritize the contribution of our input genes (kinase hits, genetic hits and differentially expressed genes) as described in the next section. The crucial insight in using this algorithm is that the derived pathways for each commodity must share the allotted capacity through each edge, which has been shown before to infer more context-specific signaling responses, because it is not allowed to send all the flow through general stress pathways. The exact problem formulation has been described previously [16].

### Inputs to SAMNet

In our network framework, each input gene hit is associated with a score that determines the maximum flow passing through that gene node. This is called an input capacity and we use these capacities to weight input gene nodes depending on the strength of evidence we have for their importance in the cellular response to gemcitabine. Below we describe how we compute these capacities.

#### Prioritization of kinase hits

The input capacities used for prioritizing kinase hits represent the maximum change in activity from one of the kinase inhibitors modifying gemcitabine cytotoxicity. If a kinase is a target (its activity changed by > 50% by the kinase inhibitor) of multiple candidate kinase inhibitors that modify gemcitabine efficacy, we use the largest score. These data are available in S3 Table. For these kinase hits, we used the gene names as mapped in the original paper [7] to HUGO symbols.

#### Prioritization of genetic hits

The input capacities used for prioritizing genetic hits are the SI, in other words, the percent change in growth upon knockdown of the gene relative to the vehicle control (the definition of the SI is given in the section above entitled “*Determining the genetic modifiers of gemcitabine efficacy* “. Genetic hits with secondary validation [8] are assigned a uniform capacity of 3. These data are available in S5 Table.

#### Prioritization of differentially expressed genes

Genes changing expression in response to gemcitabine were identified as described in the section on RNA-seq data analysis. The input capacities for these genes are the absolute value of the log fold changes in expression upon gemcitabine treatment. The input data to SAMNet are available in S8 Table.

#### Protein-protein interaction data

Our protein-protein interactions were collected from [17], with confidence scores derived using [18]. We remove P300 as it is a co-factor present at most hypersensitive peaks considered (thus providing little context-specific information) as well as UBC, which similarly has pervasive protein-protein interactions.

#### Protein-DNA interaction data

We defined a TF-gene edge in our input transcriptional network if we found a TF binding motif in a DNaseI peak within 5 kb of the regulated gene’s transcriptional start site. We defined a TF binding motif using the MATCH software suite that identifies significant motif matches in the genome [19]. We used the collection of motifs entitled “vertebrate_non_redundant_minFP.prf”. Each edge in the TF-gene network is weighted by the match score of the motif in the context of the respective gene. These data are provided in S9 Table.

### SAMNet parameter optimization

SAMNet has 1 variable parameter, called gamma, which represents the tradeoff between the amount of flow sent through the network and the cost paid for including a new edge in the network. Thus, increasing gamma results in a larger network.

To optimize the tuning parameter gamma, which balances the tradeoff between small networks with high specificity and large networks with high sensitivity, we ran SAMNet for multiple gamma values. We sought a gamma value at which networks are both robust and biologically informative. To optimize gamma, we experimented with gamma values 14, 16, 18, 20 and 22.

To quantify robustness, for each commodity, we left out a fraction of the original inputs (20%) while keeping the inputs for the remaining commodities fixed, and compared the resulting fractional networks to the original one, in terms of sensitivity (number of edges from the original network captured in the fractional network) and specificity (number of edges from the fractional network that were in the original network).

Based on this analysis, we chose an optimal gamma value of 20, which provided networks with the best combined sensitivity/specificity (S2 Fig shows the mean sensitivity and specificity we get as a function of the gamma parameter).

To optimize the edge capacity controlling the maximum amount of flow per edge, we sought to identify an edge capacity that would increase the connectivity of network nodes to the experimentally validated genetic hits (i.e. would increase evidence scores), while still maintaining a good performance in terms of sensitivity/specificity. We used an edge capacity of 0.005.

### Network visualization and enrichment analysis

We visualize networks using Cytoscape 3.0 [20] and compute functional enrichment using the David website, specifically for the enrichment in GO Biological Process [21].

### Computation of node significance

We use a randomization scheme to assign p-values to network nodes, similar to [22]. We use two criteria for selecting targets: i) statistical significance and ii) biological support, as detailed below.

First, to quantify statistical significance in our network, we compute for each node a flow p-value defined as the probability of seeing a flow of the same or higher magnitude when the algorithm is run on random inputs. To this end, we ran SAMNet on 100 random inputs in a commodity-specific way, that is, we first run 100 instances where we randomize the inputs for the genetic commodity and keep the original kinase commodity inputs fixed, and then respectively randomize the inputs for the kinase commodity, keeping the original genetic commodity inputs fixed. Then, for each node, we compute a p-value for the rank-normalized flow separately for each commodity passing through the node.

A table of flow p-values for each node in the network can be found in S12 Table. A value of -1 is given if the node does not appear at all in the random networks, denoting that no p-value is available for it. A value of NA denotes nodes that were the original input to SAMNet, and were thus not considered in this analysis.

Second, to quantify biological support for putative targets, we compute for each node an evidence score, defined as the number of inputs (genetic hits or kinase hits) upstream of the node in our network. A high evidence score corresponds to a node included in the network due to multiple lines of experimental evidence.

To be considered significant, a node must: i) have a flow-pvalue < = 0.05 (before multiple hypothesis testing correction) and ii) have an evidence score higher than or equal to 3.

## Results

### Identifying kinase inhibitors that modify gemcitabine cytotoxicity in pancreatic cancer cells

**Fig 1:**
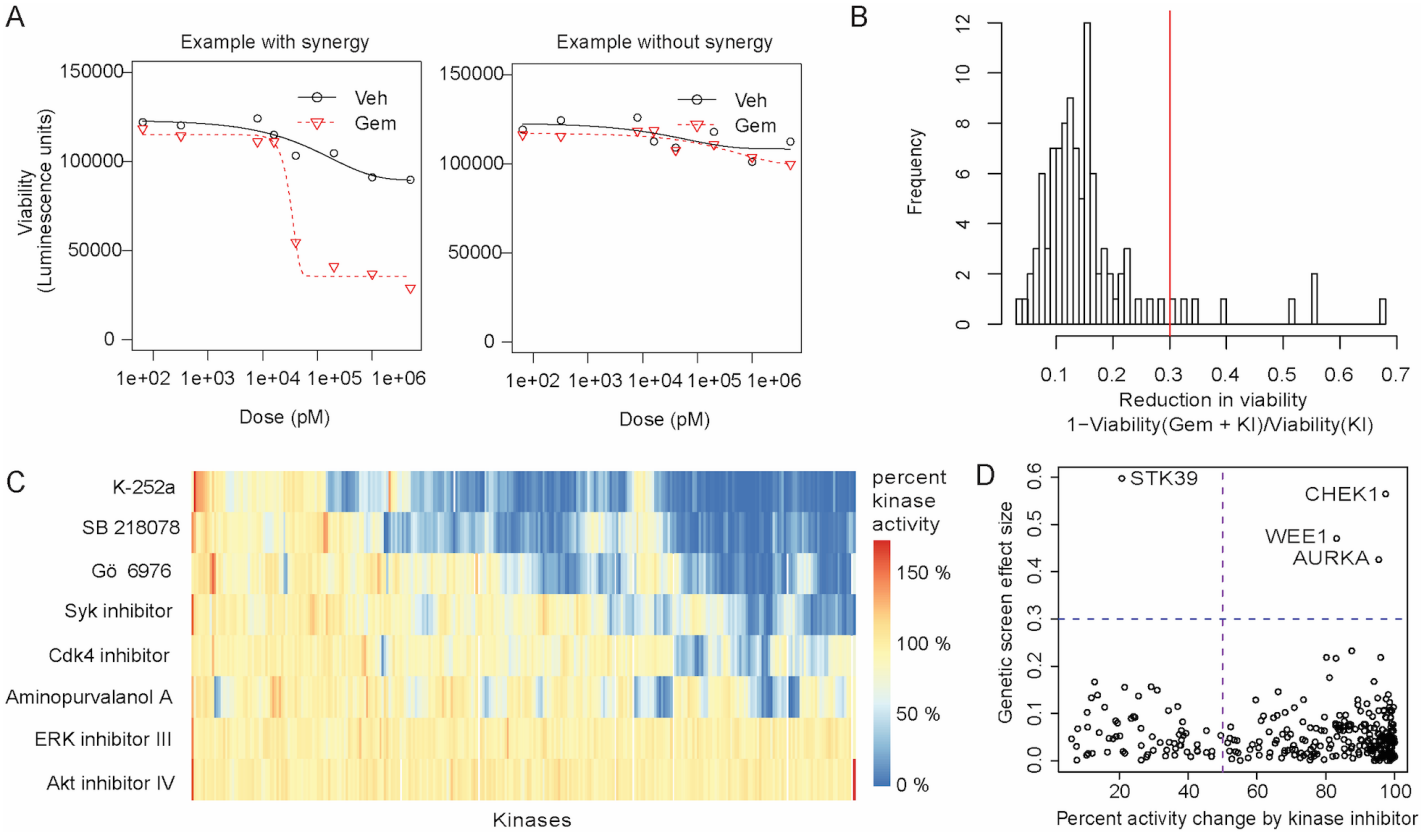
A screen to identify kinase inhibitors that modify gemcitabine cytotoxicity in pancreatic cancer (PANC1 cell line). We performed a screen to identify kinase inhibitors that enhanced killing of PANC1 cells by a sublethal dose (20nM) of gemcitabine, with hits defined as those kinase inhibitors that reduce survival by > 30% when combined with gemcitabine versus a vehicle control. A) For a set of 160 kinase inhibitors, we compare growth of PANC1 cells treated with the kinase inhibitor alone (vehicle) with the growth when treated with the kinase inhibitor and gemcitabine (Gem), across 8 kinase inhibitor concentrations. We show examples of a kinase inhibitor that synergizes with gemcitabine and one that does not. B) Identification of kinase inhibitors that modify gemcitabine cytotoxicity. For each kinase inhibitor and each concentration tested, we compute the reduction in viability for cells treated with both a kinase inhibitor and gemcitabine, compared to cells treated with the inhibitor alone. We filter out kinase inhibitors that are toxic in the absence of gemcitabine. We set a threshold of 30% reduction in viability for calling hit kinase inhibitors (red line). Note: for each kinase inhibitor, we only plot here the concentration tested that yields the largest reduction in viability. C) Mapping of kinase inhibitors to their target kinases, based on in vitro profiling of kinase inhibitor specificity from [7]. Rows are kinase inhibitors from the screen and columns are kinases. The values in the heatmap represent the percent activity of the kinase when treated with the kinase inhibitor, compared to when untreated. D) Comparison of effect sizes in the kinase screen vs. the genetic screen. For each target of our hit kinase inhibitors, we show its change in activity by the kinase inhibitor vs. the change in gemcitabine survival when the gene is inhibited by siRNA in [8] labeled as “Genetic screen effect size”.

We performed a small molecule screen to identify kinase inhibitors that enhanced killing of pancreatic cancer cell line PANC1 by a sublethal dose (20nM) of gemcitabine (Fig 1A). We defined our hits as those kinase inhibitors that produced a reduction in survival by more than 30% when combined with gemcitabine versus when combined with a vehicle control.

We filtered out kinase inhibitors that were toxic in the absence of gemcitabine treatment. Of the 160 kinase inhibitors tested, eight showed synergy with gemcitabine: Aminopurvalanol A (catalog number 164640), Syk inhibitor (574711), Cdk4 inhibitor (219476), Akt inhibitor IV (124011), SB 218078 (559402), ERK inhibitor III (328009), K-252a Nocardiopsis sp. (420298) and Gö 6976 (365250) (Fig 1B) (S1 Table).

We then sought to understand the molecular pathways targeted by each kinase inhibitor that enhanced gemcitabine efficacy. For this, we took advantage of previously published work [7] that identified kinases whose activity is changed (reduced or increased) more than 50% by these eight small molecules (S2 Table) (Fig 1C). We will refer to these affected kinases as kinase hits, as they are directly targeted by the kinase inhibitor hits from our small molecule screen. Of the kinases that are affected by these drugs, 201 are expressed in PANC-1 cells. Surprisingly, the ERK inhibitor III did not affect any of the kinases in this assay that are expressed in PANC1. The lack of targets for this molecule could be due to the following technical reasons. First, only 87% of all human kinases were tested in [7], and the targets could have fallen in the remaining 13%. Second, in many instances, the kinases in the panel are not full length; as a result, inhibitors that act in an allosteric manner may be deemed ‘inactive’, although in the cellular context they may be affected by the kinase inhibitor. In contrast to the ERK inhibitor, some of the kinase inhibitors are highly promiscuous, including K-252a Nocardiopsis sp., SB 218078, and Gö 6976, each of which target more than half of the kinases. Although a few kinases are targeted by multiple inhibitors, no clear pattern emerges that explains the inhibitors’ chemosensitization effect.

Since the biochemical data alone do not provide a clear picture of the pathways that sensitize cells to gemcitabine, we examined previously published genetic data to get a more complete picture of the mechanisms of gemcitabine sensitization. Specifically, we compared the kinase targets discussed above to a recent genome-wide siRNA experiment that identified numerous genes that, when silenced, led to modified sensitivity of PANC1 cells to gemcitabine treatment (at 50 nM gemcitabine, corresponding to an IC20) [8]. This study identified 212 genes whose knockdown alters gemcitabine sensitivity, and we term these genes genetic hits (S4 Table). We reasoned that while both the genetic and chemical screens have limitations, any overlapping targets between the two screens should provide high confidence hits. Although the kinase inhibitor screen and the siRNA screen tested for effects on the same phenotype of reduced cell survival, they had only 3 hits in common (of the 201 kinase hits and 212 genetic hits) (Fig 1D). The three genes that were revealed in both screens were WEE1, a tyrosine kinase that has previously been found to synergize with gemcitabine in a subset of pancreatic cancer xenografts [23], CHEK1, a key-regulator of cell-cycle that has been implicated in gemcitabine resistance in some pancreatic cell lines [24] and AURKA, a kinase involved in mitotic spindle function. Both CHEK1 and WEE1 passed a second round of stringent siRNA validation in [8]. Moreover, in an analogous kinase inhibitor screen, both WEE1 and CHEK1 were similarly identified as kinases whose activity when inhibited resulted in enhanced sensitivity to gemcitabine [6]. These independent studies suggest that both WEE1 and CHEK1 are bona fide enhancers of gemcitabine toxicity. However, while we observed a synergistic effect with inhibition of AURKA with gemcitabine, inhibition of AURKA was toxic even in the vehicle treatment, suggesting is it an essential gene and so it was not selected for secondary validation testing in [8].

To determine if the sensitization achieved by the eight small molecules identified in the kinase inhibitor screen could be fully attributed to CHEK1 and WEE1, we used the published kinase-target dataset to determine which small molecule affected which proteins. We found that four of the eight kinase inhibitors that enhanced gemcitabine cytotoxicity inhibited CHEK1: Syk inhibitor, SB 218078, K-252a Nocardiopsis sp. and Gö 6976. SB 218078 also inhibited WEE1. Therefore, it appears that four of the eight small molecules that enhanced sensitivity to gemcitabine may act, at least in part, by targeting CHEK1 and, in the case of SB 218078, also WEE1.

Although CHEK1 and WEE1 offer a parsimonious mechanism for enhanced sensitivity to gemcitabine achieved by four of the identified kinase inhibitors, they fail to explain the action of the remaining four small molecules, suggesting the existence of additional mechanisms for sensitization. Moreover, the example of SB217078, which targets both CHEK1 and WEE1, suggests that efficacy can be achieved by inhibiting multiple kinases at once that have combined downstream effects. Previous work shows examples of this in the context of gemcitabine-kinase inhibitor interactions [25], emphasizing the need for integrative methods to infer the underlying pathways of synergy with gemcitabine by leveraging information across the eight small molecules. Finally, a recent study to identify synergizers with gemcitabine in PANC1 cells has shown CHEK1-independent mechanisms that result in cellular death [8], further supporting our assumption of additional pathways of gemcitabine sensitization beyond CHEK1 and WEE1.

### Profiling of the gemcitabine response by RNA-seq and DNaseI-seq reveals complementary information to the kinase inhibitor screen

To accurately model the mechanisms of enhanced gemcitabine sensitivity elicited achieved by each of the eight identified small molecules, we set out to leverage information from complementary experiments that examine the response of pancreatic cancer cells to gemcitabine. For instance, gene expression measurements could reveal which small molecule targets (and which of the kinases’ substrates) are expressed, reducing the search space of predicted kinase targets to those that are active in pancreatic cancer. In addition, measuring changes in the epigenome combined with knowledge of gene expression changes in response to gemcitabine can identify transcription factors (TFs) that are activated or repressed to achieve gemcitabine-induced cell death, allowing us to prioritize kinases that influence these TFs. The importance of transcription factors in gemcitabine resistance is highlighted by a recent study [8] that identified the transcription factor vitamin D receptor (VDR) as a mediator of gemcitabine chemoresistance, through a novel mechanism that affected the homologous recombination repair pathway.

**Fig 2:**
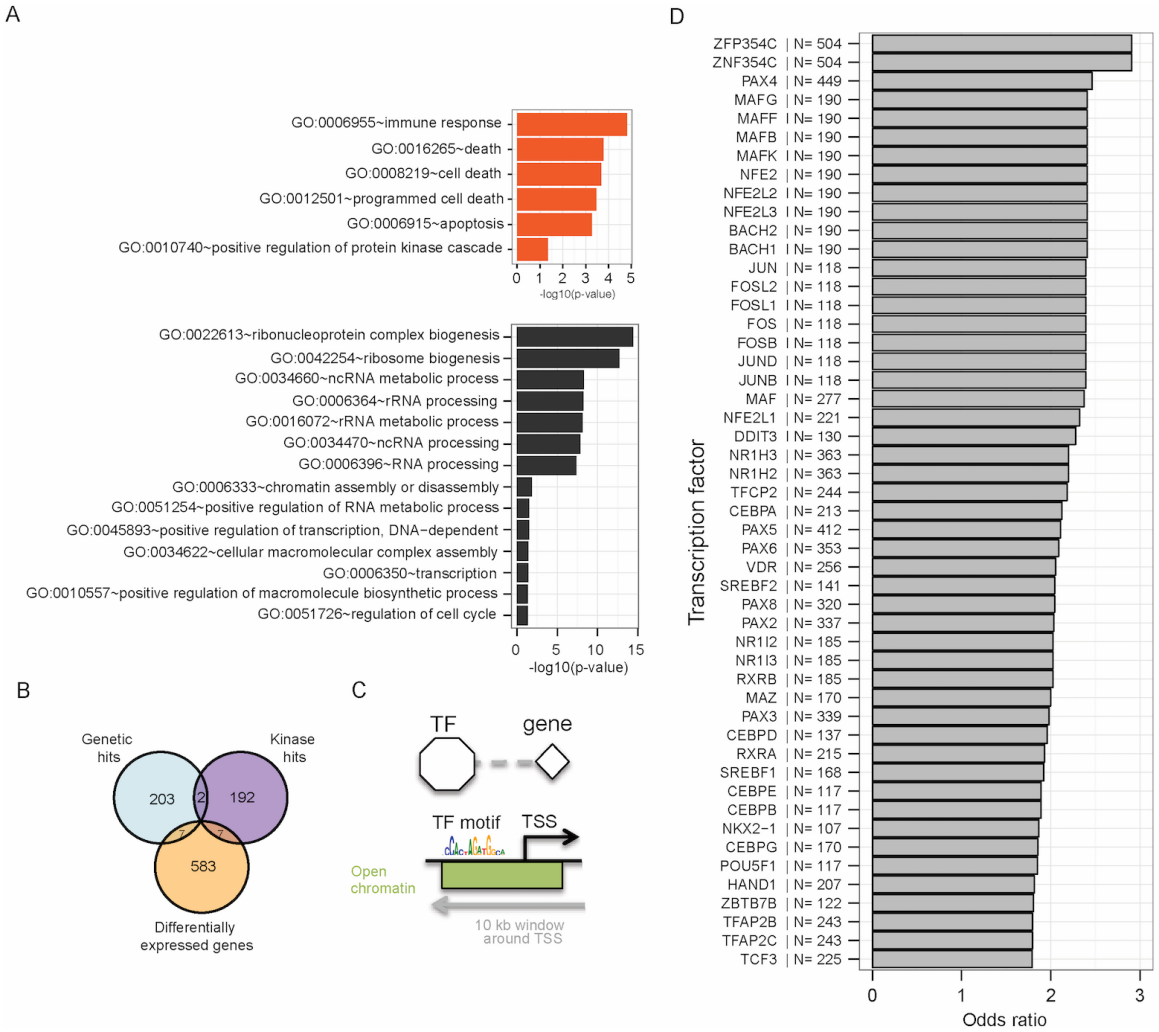
Profiling the transcriptional and epigenomic response to gemcitabine. We measured changes in gene expression upon treatment of PANC1 cells with gemcitabine by RNA-seq. To understand the gene regulatory changes in response to gemcitabine treatment, we profiled DNaseI hypersensitivity for gemcitabine and vehicle-treated PANC1 cells. A) GO enrichment analysis for genes changing expression in response to gemcitabine (at the top in orange are genes up-regulated upon drug treatment, below in black are the genes down-regulated). B) Overlap between the genes that change expression when treated with gemcitabine (differentially expressed genes), genetic modifiers of the gemcitabine resistance (genetic hits) and targets of our hit kinase inhibitors that sensitize cells to gemcitabine (kinase hits). We find a modest overlap between the three sets, a trend observed before when comparing complementary high-throughput profiling approaches [27]. C) Construction of a TF-DNA regulatory network using DNaseI hypersensitivity data collected for cells treated and untreated with gemcitabine. We called peaks on the DNaseI data (combined with existing data for the same cell line from the ENCODE project), and then scanned each peak for TF binding sites using Transfac motif matrices. We assign a TF-gene regulatory interaction if we find a TF motif in a DNaseI peak that is within 5kb of the gene’s transcription start site. D) Top 50 transcription factors enriched in the promoters of differentially expressed genes. For each transcription factor, we performed a Fisher’s Exact Test to ask whether we see an overrepresentation of the transcription factor’s associated motifs in the promoters of genes changing expression in response to gemcitabine, compared to its presence in all promoters harboring a DNaseI peak. Note: we show here only those TFs with motifs in promoters of more than 100 differentially expressed genes.

We measured changes in gene expression upon gemcitabine treatment in pancreatic cancer cell line PANC1 by RNA-seq (see Methods). We sequenced two biological replicates treated with gemcitabine, and two untreated replicates, each at ~20 million reads. We identified 428 genes up-regulated upon gemcitabine treatment and 169 down-regulated genes (S6 Table). As expected, genes that are activated are enriched for processes related to cellular death, including programmed cell death, consistent with the apoptotic effect induced by gemcitabine (Fig 2A) (S7 Table) [21]. We also found enrichment for genes related to the “immune response” category, as differentially expressed genes include a set of interleukins (IL27RA, IL18, IL32, IL10RB, IL1B, IL6, IL8), as well as immune-related transcription factors such as NFKB2. Finally, genes down-regulated upon gemcitabine treatment are enriched for RNA processing and cell cycle (Fig 2A). The down-regulation of genes important for RNA processing pathways is consistent with previous work showing down-regulation of ribosomal RNA during gemcitabine treatment [26].

The genes that change in expression are mostly distinct from the genetic hits and the kinase hits previously identified (Fig 2B), a trend known to arise when comparing multiple ‘omics datasets describing the same system [27]. The set of genes identified by more than one assay are i) the genes that are both genetic hits and kinase hits CHEK1 and WEE1 described above; ii) the genetic hits that change expression upon treatment with gemcitabine HINT2, C19orf48, PLXDC2, ZFYVE19, IGFBP6, SCPEP1, ERRFI1 and iii) the kinase hits that change expression upon treatment with gemcitabine: CAMKK1, EPHA7, MAP4K2, MAP2K6, PDGFRB, NUAK2, DAPK3 (Fig 2B).

To effectively compare transcriptional changes with the pathways that give rise to gemcitabine sensitization, we sought to identify specific transcriptional regulators that could explain the observed changes in gene expression. To do so, we used epigenetic data to identify transcription factors that may bind in open chromatin regions nearby differentially expressed genes. Specifically, we identified open chromatin regions by using DNaseI-seq under the same conditions as those for the siRNA screening and RNA-seq (see Methods), and supplemented these regions with those derived from existing ENCODE DNaseI hypersensitivity data in the same cell type [15]. In total we identified 117,904 open chromatin regions in PANC1 cells. We then scanned these regions for matches to transcription factor binding motifs from the TRANSFAC database [28], and assigned TF-gene regulatory interactions if a motif match was identified within 5kb of a differentially expressed gene’s transcription start site (TSS) (Fig 2C). Given this TF-gene network (S9 Table), we asked which TF motifs were enriched in the promoters of the genes differentially expressed in response to gemcitabine. Fig 2D shows the top 50 enriched TFs. We find the top enrichment for ZFP354C, ZNF354C, PAX4, MAF-related proteins and NFE2-related proteins. The set of enriched TFs also includes TFs previously reported to contribute to the response to gemcitabine including VDR and RXRA [8]. Finally, some TFs from the collection are genetic hits, as is the case for RFXAP, RUNX2, TBX5, SRF and VDR.

Equipped with four distinct datasets profiling either the response to gemcitabine (gene expression and epigenomic changes) or pinpointing key players in gemcitabine sensitization (kinase hits and genetic hits), we set out to build a network model that integrates the different datasets, and generates hypotheses for mechanisms of gemcitabine sensitization. We had two goals: i) to identify the multiple pathways by which the eight synergizing kinase inhibitors may work and ii) to produce a restricted set of genes amenable to future studies of gemcitabine sensitization.

### Using the SAMNet algorithm to link the transcriptional and epigenetic changes to upstream genetic and kinase hits through the protein-protein interaction network

**Fig 3:**
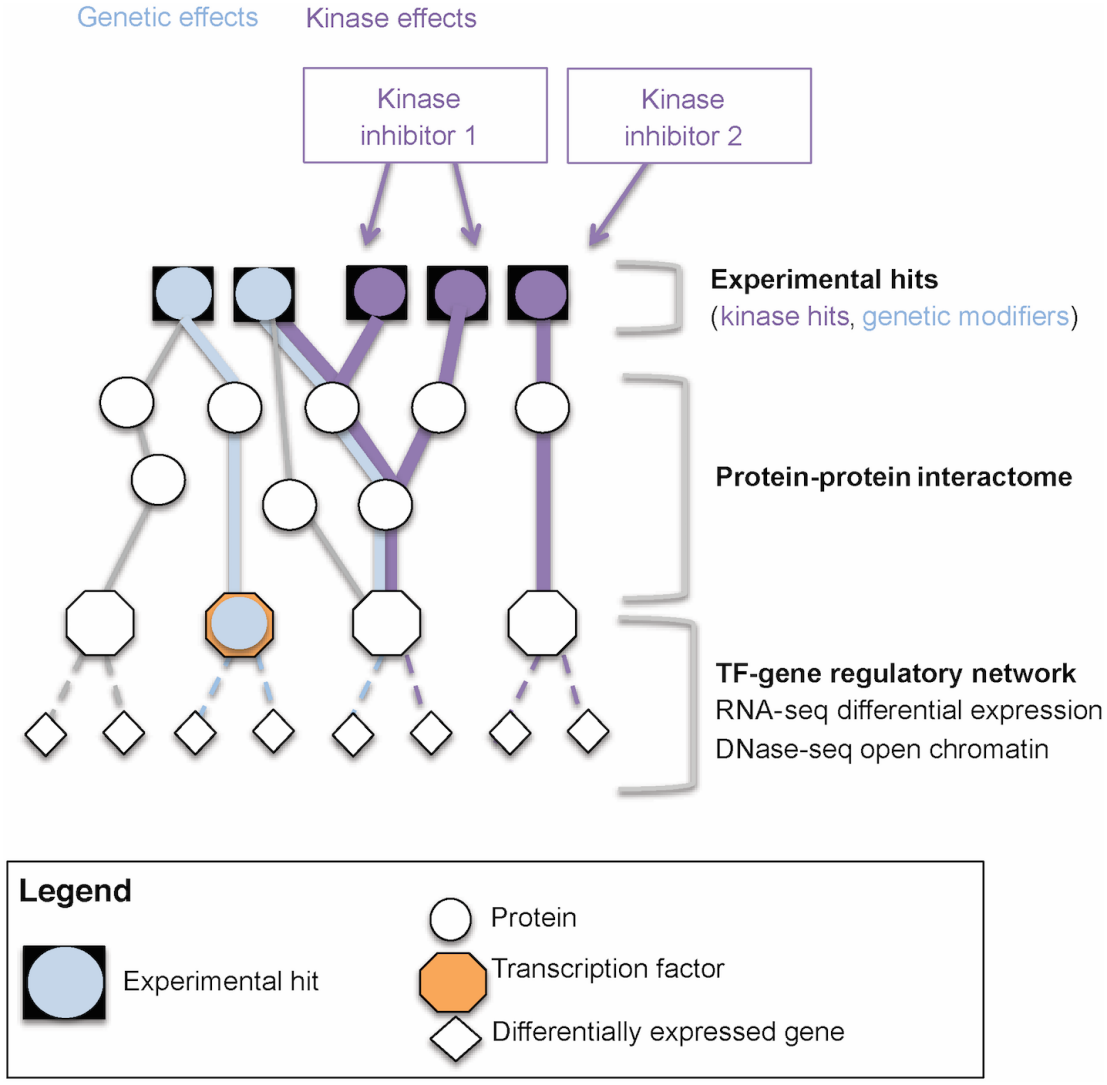
General approach for data integration using the SAMNet algorithm. We used the SAMNet algorithm to model the cellular response to gemcitabine. SAMNet uses as inputs i) kinases that are predicted to be targeted by the eight gemcitabine-synergizing kinase inhibitors discovered, ii) genetic modifiers of gemcitabine efficacy (genetic hits), iii) genes changing in expression upon gemcitabine treatment, and iv) a TF-gene network based on TF motif matches in open chromatin regions in the promoters of the differentially expressed genes. The SAMNet algorithm then connects the input kinases and genetic hits to the differentially expressed genes through the protein-protein and the TF-gene interactomes in a constrained optimization setting. To distinguish networks anchored in kinase target hits from those anchored in genetic hits, we defined two optimization problems (or commodities) that are solved simultaneously. These are depicted as purple and blue.

We integrated the four datasets into a network model using the SAMNet algorithm [16]. Our goal was to identify biological pathways that connect the experimentally determined genes of interest via reported protein-protein interactions and predicted TF-gene interactions. This approach, summarized in Fig 3, relies on published protein-protein interaction data to provide physical links between the distinct proteins implicated in gemcitabine sensitization given the screens described above. Our protein-protein interactions were collected from [17] and scored using [18]. The TF-gene interactions were predicted using the epigenetic data as described above (Fig 2C).

The algorithm starts with the kinase hits and genetic hits and uses a protein-protein interactome to link these hits to the transcription factors predicted to regulate genes differentially expressed upon gemcitabine treatment. The transcription factor genes are further connected to their putative target genes through the TF-gene regulatory interactions defined in the previous section. The SAMNet algorithm identifies pathways of interest by finding a subset of protein-protein and TF-gene interactions that connect the kinase hits and genetic hits (squares, Fig 3) to differentially expressed transcripts (diamonds, Fig 3). To simultaneously model both the effects of genetic hits and the effects of kinase hits we defined our input set of genes as two groups: a group of genetic hits and a group of kinase hits and then treated each group as independent “commodities” that must share the same paths through the network. Kinase hits were linked to their putative targets using the data from [7]. The algorithm tries to connect as many experimental inputs to the transcriptional changes while minimizing a penalty cost paid for each edge included in the network, proportional to the prior evidence of the interaction represented by that edge; this approach provides a sparse network that is focused on the molecules and interactions with the strongest support. Since this optimization is simultaneously performed for the genetic hits and the kinase hits, we obtain a two-commodity network, specifying for each included node and edge the proportion of commodity allocated for each edge. After removing those experimental hits that were not present in the protein-protein interaction network, we ran SAMNet with 171 kinase hits (S3 Table), 138 genetic hits (S5 Table), and 532 differentially expressed genes (S8 Table). We obtained a network with 697 protein nodes connected through 831 edges (S1 Fig) (S10 Table), of which 115 nodes are shared between the genetic and the kinase commodities.

We then proceeded to check the network predictions in terms of i) biological processes overrepresented in the network and ii) specific genes included in the network. We performed a global GO enrichment analysis specifically on the nodes identified by SAMNet, while excluding our input nodes (genetic hits, kinase hits, differentially expressed genes). The main classes of biological processes enriched in our network (S11 Table) are: i) phosphorylation (p-value corrected for multiple hypothesis testing using the Bonferroni correction: 1.40E-08), ii) transcription (p-value: 2.13E-41), iii) apoptosis, cell cycle (p-value: 1.10E-17), iv) response to DNA damage (p-value: 2.48E-04), DNA repair (p-value: 0.02411934), DNA damage checkpoints (p-value: 0.011921177), v) proliferation, cell growth (p-value: 3.31E-17), vi) response to organic substance (p-value: 8.79E-13), response to radiation (p-value: 0.005290541), and vii) development (p-value: 5.10E-08). Some of these terms are to be expected, such as (i) and (ii), since our network is anchored on kinases on one end, and on transcription factors on the other end. However, the additional biological processes reveal a more comprehensive view of the identified network.

**Fig 4:**
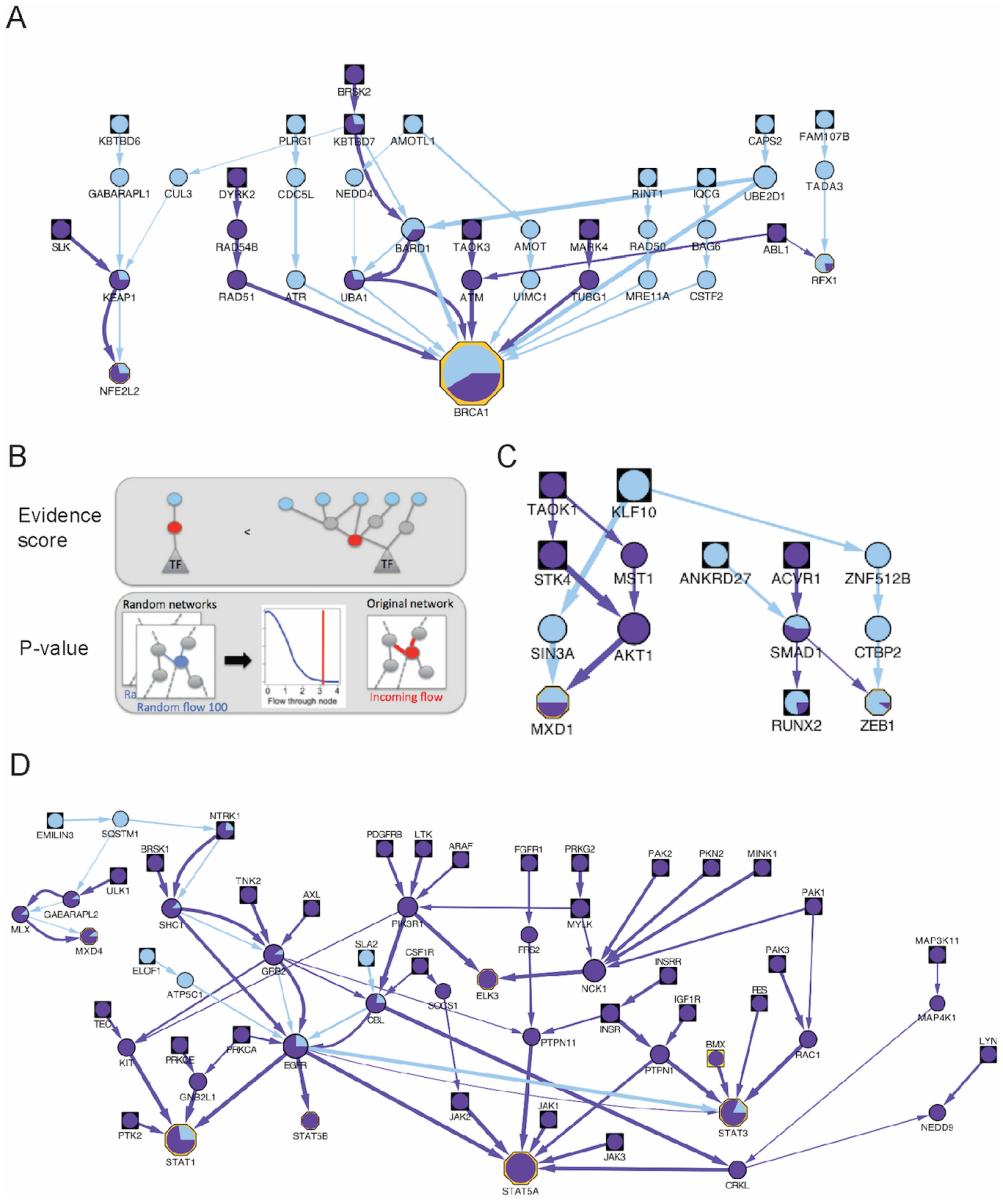
SAMNet network results. For displaying network results, we use the following graphical strategy. Node size scales with the flow passing through each node. Nodes are colored based on the proportion of flow they get from the two commodities: kinase hit (purple) and genetic hit (blue). Edge-width scales with the flow passing through each edge, and the color represents the commodity type (kinase or genetic hit). The node shape specifies whether the node is a TF (octagon), and whether it is an experimental input gene (square). A) Example subnetwork identified using SAMNet, involved in the DNA damage response. B) Strategy for identifying significant nodes from the network. We use two metrics to identify the most meaningful genes from the network: i) evidence score counting how many experimental inputs are connected to the gene and ii) a node p-value based on comparing the observed flow a node receives to the expected flow received in a set of 100 networks with random inputs. C) Subnetwork containing the significant nodes AKT1, MXD1, ZEB1 identified using our network method. D) Same as C), but containing significant nodes SHC1, ELK3, STAT5A, NCK1, PTPN1.

The enrichment for apoptosis, proliferation and DNA damage response, for example, supports the validity of our network, since gemcitabine’s mechanism of action involves apoptosis as a result of DNA damage. Indeed, analysis of subnetworks (Fig 4A) recovers the main pathway involved in gemcitabine action, DNA damage response. As an example, the cluster containing BRCA1 (Fig 4A) captures two distinct methods of responding to DNA damage. In this subnetwork we find mechanisms both for promoting apoptosis upon DNA damage and for escaping apoptosis. For instance, proteins involved in the S-phase checkpoint including ATR, BRCA1 and CDC5L support the action of gemcitabine, by induction of cell-cycle arrest and apoptosis upon DNA damage [29–31]. On the other hand, we also find proteins involved in double stranded DNA repair that in theory counters the effect of gemcitabine. For instance, RAD50 and MRE11A form a complex active in DNA damage repair by recombination [32].

Additionally, several subnetworks recover known pathways of gemcitabine resistance including the epithelial to mesenchymal transition and histone deacetylation (S1 Fig). The epithelial to mesenchymal transition is manifested through proteins of the beta catenin pathway, a pathway previously reported to distinguish gemcitabine resistant from sensitive cells [33,34]. The role of histone deacetylation [35] is highlighted by four HDACs in our network, and confirmed by previous reports of gemcitabine sensitization upon combination treatment with histone deacetylase inhibitors [36].

Finally, we also find enrichment for development, differentiation and lymphoid and immune-related development. The enrichment for immune system development is in large part due to TFs and regulators/co-activators included in this GO category, such as STAT factors, PPARG, PML, NFKB2, TP53, RB1, SP1, TCF3, CEBPA, HOXA9, BCL6, EGR1, NCOA6 and SCAND1. Some of the genes in this category have been previously implicated in gemcitabine resistance and pancreatic cancer, suggesting that our network identifies highly meaningful gene sets for the cellular response to gemcitabine. For instance, NFKB2 (described in more detail the next section) is important for gemcitabine sensitization: inhibiting NFKB2 increases the gemcitabine cytotoxicity in pancreatic cancer cell lines BxPC2, Capan1 and PancTu1 [37].

### Highly ranked network nodes modify gemcitabine efficacy and suggest mechanisms of chemosensitization

We next sought to identify particular nodes in the network that would be of greatest relevance to gemcitabine sensitization. We began by assessing the statistical significance of nodes in our network. The network optimization method that we used is known in the computer science literature as “maximum-flow-minimum-cost;” the flow is an abstraction that is related to the importance of a node in connecting the hits from the screen to the differentially expressed genes. See reference [27] for details. To assess the significance of nodes we asked which nodes receive more flow than would be expected by chance (see Methods, Fig 4B). For this purpose, we generated 100 permuted inputs for the genetic commodity, and 100 permuted inputs for the kinase commodity, and ran SAMNet on each. This produced a distribution of values for each node in our original network representing the ‘flow’ that the algorithm allocates to the incoming and outgoing edges. As flow corresponds to the relative importance of that node in the overall network, we computed a p-value that represents the probability of achieving, under the null hypothesis, a flow equal or higher than the one observed in the real network. We then compared the significance of each node with the number of experimental hits connected to the node (directly or indirectly), which we refer to as the evidence score (Fig 4B). Selecting for targets with a flow p-value < 0.05 and an evidence score > 3 (S12 Table), we identified a set of putative targets for increasing pancreatic cancer killing upon gemcitabine treatment. Table 1 shows the 23 targets identified in this manner.

**Table 1:** Candidate gene targets from SAMNet.

Node	Evidence score	Pvalue (genetic)	Pvalue (kinase)
ZBTB7A	40	0.99	0.04
STAT5A	31	1	0.01
NKX2-1	16	1	0.02
ELK1	11	0.97	0.01
ELK3	9	0.99	0.01
NFKB2	8	0.01	0.89
TGIF1	8	0.31	0.01
IRF7	6	0.01	0.19
NCK1	6	0.97	0.01
TNFRSF11A	4	0.01	0.9
TRAF6	4	0.05	0.35
MUC1	4	0.99	0.05
MXD1	4	0.08	0.03
TLX2	3	0.01	0.84
YWHAH	3	0.01	0.24
ZEB1	3	0.04	0.07
AKT1	3	0.99	0.05
ZNF350	3	0.01	0.05
CRK	3	0.23	0.01
NFATC2	3	0.99	0.01
PRKCZ	3	0.98	0.01
PTPN1	3	0.99	0.01
SHC1	3	0.26	0.01

Several of the top targets are strongly supported by prior data. Among the significant network nodes 14/23 were supported by literature to be involved in pancreatic cancer (ELK1, ELK3, NFKB2, MXD1, NCK1, AKT1, YWHAH, STAT5A, TRAF6, MUC1, ZEB1, NFATC2, PRKCZ and SHC1). 8 additional genes, CRK, ZNF350, ZBTB7A, TGIF1, PTPN1, NKX2-1, IRF7 and TNFRSF11A have been linked to other cancers. Of these targets supported by literature to be involved in pancreatic cancer, 9/14 were previously implicated in gemcitabine resistance: NFKB2, AKT1, ZEB1, NFATC2, SHC1 and MUC1 modify gemcitabine resistance in pancreatic cancer, while ELK1 increases gemcitabine cytotoxicity in prostate cancer but has also been studied due to its transcription regulatory effect in pancreatic cancer. STAT5A, TRAF6 are over-expressed in pancreatic cancer cells resistant to gemcitabine [38]. In addition, of the genes involved in other cancers, 2/8 are related to gemcitabine: TGIF-1 modifies gemcitabine resistance in bladder cancer [39], and NKX2-1 higher expression increases gemcitabine cytotoxicity in lung cancer cells [40].

A detailed description of the literature support for the candidate genes can be found in (S13 Table). Below, we describe a few examples and the related hypotheses generated by our network model.

First, our network predictions include kinases expected to participate in the cellular response to gemcitabine. Among these kinases is AKT1 (Fig 4C). The effect of AKT1 inhibition on gemcitabine efficacy has been studied extensively. AKT inhibition through triciribine increases the anti-tumor efficacy of gemcitabine in a mouse xenograft model (xenografts from the human SU86 pancreatic cancer cell line), [41]. In addition, evodiamine, which targets AKT1 and PI3K, PKA, mTOR and PTEN sensitizes pancreatic cancer cells to gemcitabine [42]. On the other hand, AKT1 inhibition through arsenic trioxide (which targets a large set of genes) following gemcitabine treatment did not increase chemosensitivity in a Phase II clinical trial for pancreatic cancer [43]. Our networks include AKT1 even though it did not appear in our list of genetic or kinase hits. Interestingly, our kinase inhibitor screen did identify AKT inhibitor IV as a modifier of gemcitabine cytotoxicity, but this inhibitor works by inactivating AKT1 indirectly, through an upstream regulation mechanism [44].

Second, our network suggests additional gene products beyond kinases mediating chemoresistance to gemcitabine. For instance, one of our highly ranked nodes, NFKB2 (S1 Fig), a subunit of the NFKB transcription factor, has been previously found to be up-regulated in a dose-dependent fashion by gemcitabine, and its inhibition reduced gemcitabine resistance robustly across pancreatic cancer cell lines BxPC3, Capan1 and PancTu1 [37]. Similarly, ZEB1 (Fig 4C), a protein involved in the epithelial to mesenchymal transition already has an established role in gemcitabine resistance: its levels are inversely correlated with cellular response to gemcitabine, and silencing ZEB1 increases gemcitabine-induced apoptosis in pancreatic cancer cell lines PANC1, HS766T and Miapaca2 [34]. NFATC2, a transcription factor, when knocked down, increases the apoptosis induced by gemcitabine in PaTu-8988t pancreas adenocarcinoma cells [45]. In addition, knockdown by siRNA of adaptor protein SHC1 in pancreatic cancer cells reduces gemcitabine efficacy [46].

Our network also points to several new aspects of gemcitabine resistance in pancreatic cancer.

For instance, our network highlights NCK1 (Fig 4D), an adaptor protein that has many connections in the protein-protein interaction network. While such high-degree nodes can sometimes be false positives, our randomization strategy indicates that it is unlikely to occur in our networks by chance. NCK1 connects directly through protein interactions with four experimental hits. NCK1 has been demonstrated to affect pancreatic cancer migration but not growth [47] through EGFR, a protein to which our networks also link NCK1. Since invasiveness distinguishes gemcitabine-resistant from gemcitabine-sensitive cell lines [34], we hypothesize based on the network predictions that NCK1 affects the pancreatic cancer and gemcitabine resistance through invasiveness mechanisms.

In summary, our network approach allowed us to generate hypotheses for genes important for the response to gemcitabine, supported simultaneously by the kinase inhibitor data, genetic hits and transcriptional changes. Six of the significant nodes have already been confirmed to modify gemcitabine cytotoxicity specifically in pancreatic cancer, two more are up-regulated in gemcitabine-resistant pancreatic cancer cell lines, and five affect gemcitabine cytotoxicity in other cancers. The rest are as yet unconfirmed, and serve as starting points for future research.

## Discussion

We presented an integrative study of chemical screening data in the context of sensitization to gemcitabine chemotherapy in pancreatic cancer. We identified eight kinase inhibitors that increase gemcitabine efficacy, and studied their mechanisms of action by integrating their predicted target kinases with genetic, transcriptomic and epigenomic profiling resulting in a compact network, amenable to future detailed dynamic studies. There was little overlap among the top hits from these experiments, with no obvious common pathways that could explain the changes in gemcitabine sensitivity. However, we showed that by integrating these complementary data in a network setting such as with the SAMNet algorithm, we could recover coherent underlying biological processes.

A fundamental contribution of this study is the explicit modeling of the polypharmacology of small molecules, in our case kinase inhibitors. Many molecular therapeutics, even targeted ones, are highly promiscuous [48], and it is usually unclear which of the targets are responsible, alone or in combination, for the therapeutic benefits. Notably, the eight small molecules we found to sensitize cells to gemcitabine have been shown to affect 201 kinases. We hypothesized that more than one kinase targets of each inhibitor could contribute directly to the cellular response to gemcitabine and our network approach revealed pathways linking these proteins. Thus, the polypharmacology of those agents may sometimes be important for their function. Indeed, recent studies looking to repurpose clinical-stage drugs underscore the usefulness in exploiting the fact that small molecules affect potentially many genes at once [6,49,50]. Computational methods, such as the one presented here, provide a means to prioritize which of the hundreds of candidate kinases may affect phenotypic outcomes.

In addition to the methodological advancements, we reported key findings for understanding the response to gemcitabine chemotherapy in pancreatic cancer. First, we identified the genes that change in expression upon gemcitabine treatment in PANC1 cells using RNA-seq, allowing us to quantify more accurately changes in gene expression compared to previous microarray-based studies [51]. Moreover, our epigenomic and condition-specific DNaseI-seq profiling allowed us to trace key transcription factors that may be mediating the observed changes in gene expression. Second, our network approach recovered known modulators of gemcitabine resistance and suggested additional candidates. Finally, our network hypotheses associated these candidate genes to specific biological pathways, helping focus future studies.

To ultimately understand chemosensitization, one needs to study the intricate mechanisms of additivity, compensation and synergy between the genes involved. While this type of detailed understanding is not feasible on a genome-wide scale, it becomes tractable when focused on a subset of genes, which can be perturbed in combinations, and observed dynamically. We designed our analysis to identify precisely this subset of genes, which are now amenable to dynamic studies to elucidate the quantitative output of the network under perturbation conditions, through Boolean networks, differential equations, or QTL studies across individuals. Thus, we expect this study to serve as a starting point for future dynamic studies of gemcitabine sensitization.

Finally, we expect our method to be increasingly useful for the community in the context of the growing body of chemical screening datasets [1,2,52,53]. Kinase inhibitor screens are increasingly common, and our approach is a novel way to prioritize proteins and pathways for targeting. The fact that our method uses genetic, epigenomic and transcriptional profiling will not restrict its use, as there are publicly available datasets that can at least partially fill in this gap in other systems. For instance, baseline transcriptional data and epigenomic data are available from the ENCODE consortium [15] and the Roadmap Epigenomics consortium [54] projects. Cancer-specific RNAi and CRISPR-Cas9 screens are also available from the Achilles project [55]. Overall, our method will be a valuable tool to help interpret chemical screening data explicitly taking into account polypharmacology for improved understanding of drug action mechanisms.

## Supporting information

S1 Fig Full SAMNet network. Starting from the original obtained network, we split it into clusters using Cytoscape’s Community Cluster (GLay) clustering method.
(PDF)

S2 Fig Parameter optimization for SAMNet. To choose the value of the gamma parameter, we gamma values of 14, 16, 18, 20 and 22. For each gamma setting, we considered the networks we obtain when leaving out a fraction (20%) of the inputs. We plot as a function of gamma the combined sensitivity (number of nodes from the original network captured in the fractional network) and specificity (number of nodes from the fractional network that were in the original network) for the comparison between the SAMNet result and the networks obtained from the fractional input. Based on this, we chose an optimal gamma value of 20.
(PDF)

S1 Table Measurements for each kinase inhibitor when treating cells with gemcitabine vs vehicle control. (ZIP)

S2 Table The set of kinases whose activity is changed by more than 50% by a kinase inhibitor found to synergize with gemcitabine. (ZIP)

S3 Table Input to SAMNet: Kinases weighted by the maximum change in kinase activity by a kinase inhibitor found to synergize with gemcitabine. (ZIP)

S4 Table List of genes whose knockdown modulates the response to gemcitabine, from the siRNA screen. (ZIP)

S5 Table Input to SAMNet: Genetic hits, or genes whose knockdown modulates the response to gemcitabine, weighted by the change in growth observed with vs without the gemcitabine. (ZIP)

S6 Table Differential expression analysis for PANC1 cells with and without gemcitabine treatment. (ZIP)

S7 Table GO enrichment of differentially expressed genes. (ZIP)

S8 Table Input to SAMNet: Differentially expressed genes, weighted by the absolute value of the log fold change. (ZIP)

S9 Table Transcription factor to gene assignments, based on motif scanning in DNaseI sites in gene promoters. (ZIP)

S10 Table Obtained network from SAMNet. (ZIP)

S11 Table GO enrichment of the network obtained from SAMNet. (ZIP)

S12 Table Table containing p-values for each node in the SAMNet network based on permutations. (ZIP)

S13 Table Detailed description of literature support for candidate genes from SAMNet. (PDF)
