# Supplementary figures and images for: Network modeling of kinase inhibitor polypharmacology reveals pathways targeted in chemical screens
Source: PLoS One. 2017 Oct 12;12(10):e0185650. doi: 10.1371/journal.pone.0185650 (PMC5638242; doi:10.1371/journal.pone.0185650)

Supplementary Figure 1

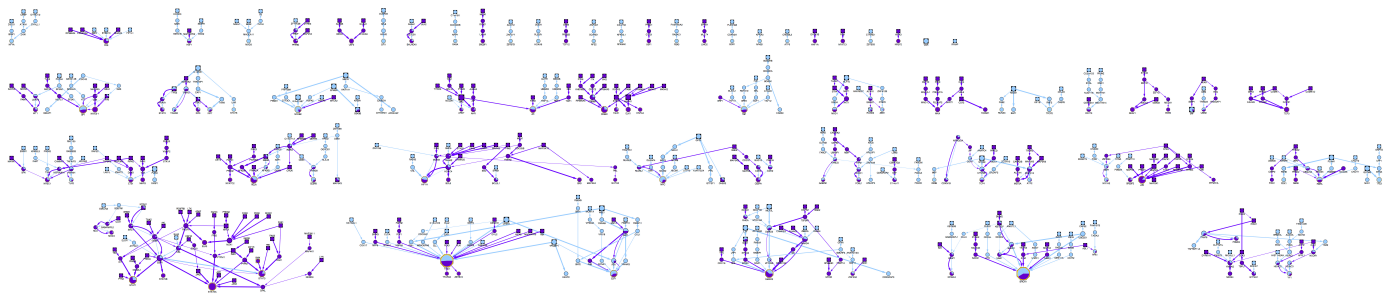

Supplement: S1 Fig — Starting from the original obtained network, we split it into clusters using Cytoscape’s Community Cluster (GLay) clustering method. (PDF) [file pone.0185650.s001.pdf]

### Gamma vs performance Network edges without mRNA

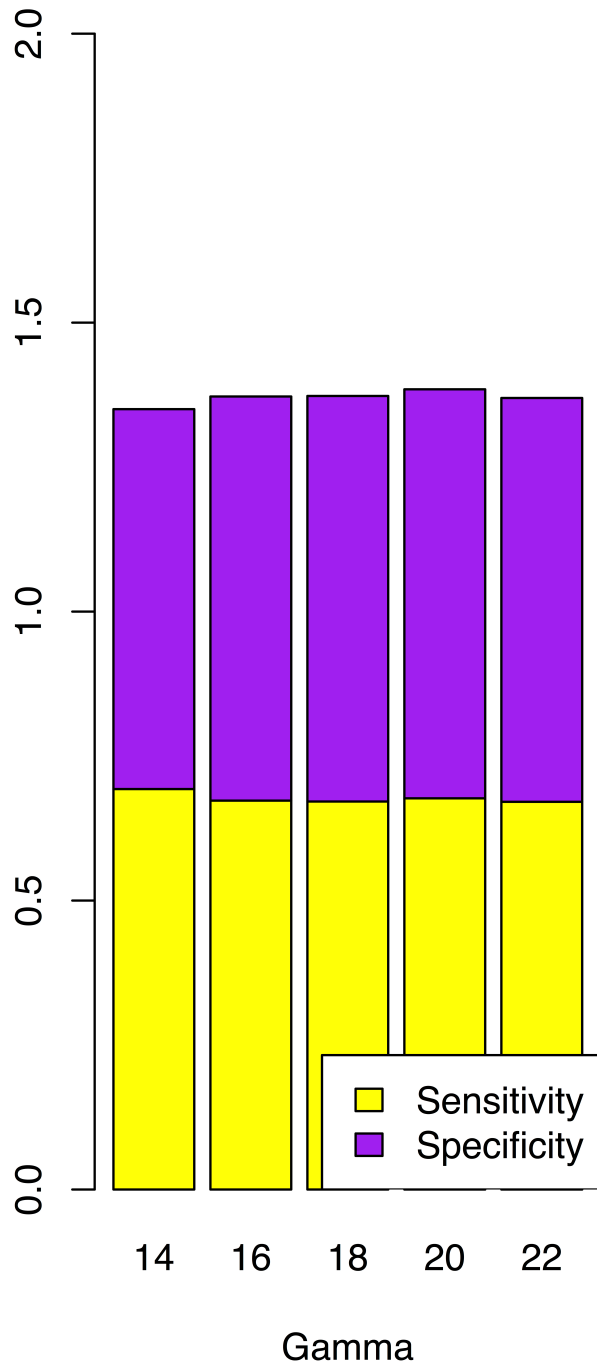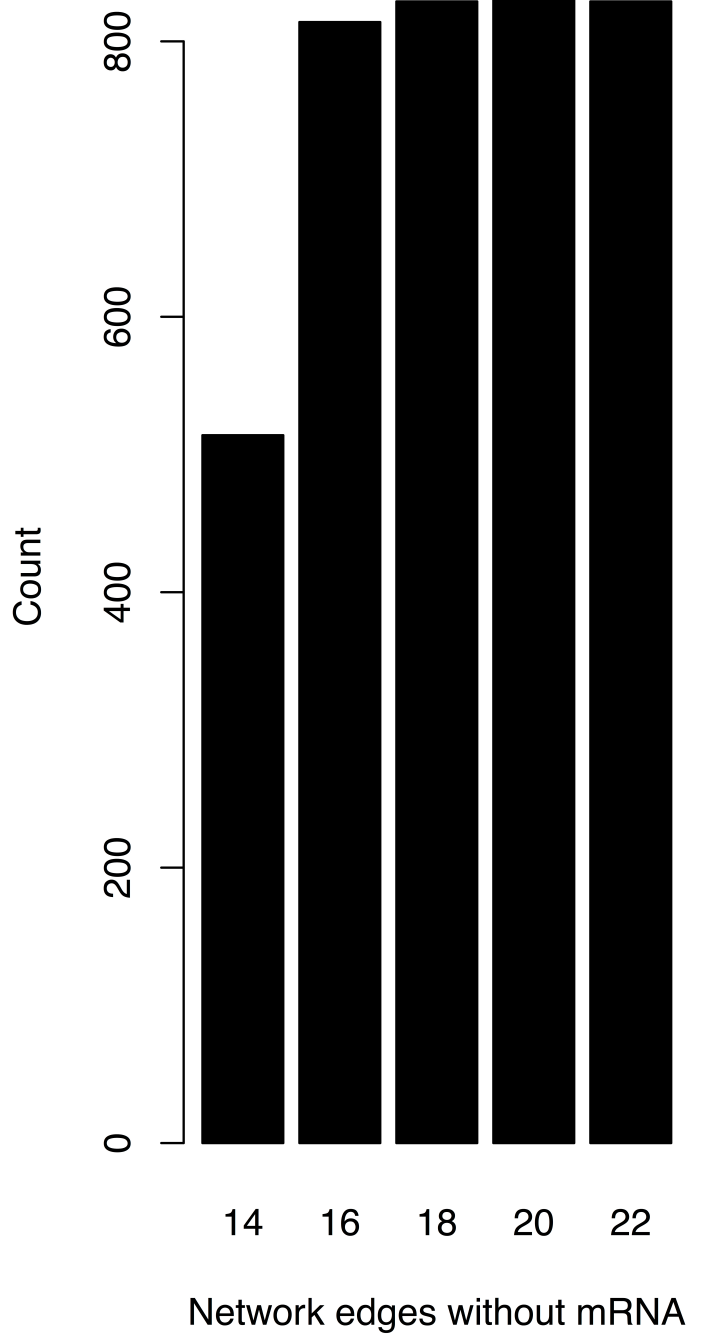

Supplement: S2 Fig — To choose the value of the gamma parameter, we gamma values of 14, 16, 18, 20 and 22. For each gamma setting, we considered the networks we obtain when leaving out a fraction (20%) of the inputs. We plot as a function of gamma the combined sensitivity (number of nodes from the original network captured in the fractional network) and specificity (number of nodes from the fractional network that were in the original network) for the comparison between the SAMNet result and the networks obtained from the fractional input. Based on this, we chose an optimal gamma value of 20. (PDF) [file pone.0185650.s002.pdf]
