# Supplementary material for: Network modeling of kinase inhibitor polypharmacology reveals pathways targeted in chemical screens
Source: PLoS One. 2017 Oct 12;12(10):e0185650. doi: 10.1371/journal.pone.0185650 (PMC5638242; doi:10.1371/journal.pone.0185650)
Supplement: S13 Table — (PDF) [file pone.0185650.s015.pdf]

Gene	Gene description	Finding	Organism/ cell line	Involved in gem response?	Involved in pancreatic cancer?	Citation
AKT1	Kinase	AKT1 inhibition through triciribine combined with gemcitabine leads to increased cell death compared to gemcitabine alone.<br><br>Evodiamine, which targets AKT1 and PI3K, PKA, mTOR and PTEN sensitizes pancreatic cancer cells to gemcitabine.<br><br>AKT1 inhibition through arsenic trioxide (which targets a large set of genes) following gemcitabine treatment did not increase chemosensitivity in a Phase II clinical trial for pancreatic cancer	Mouse xenograft (xenograft from the human pancreatic cancer cell line SU86)<br><br>Pancreatic tumors implanted in mice<br><br>Clinical trial	Yes	Yes	[1]<br><br>[2]<br><br>[3]
NFKB2	Subunit of NFKB transcription factor	Inhibiting NFKB2 increases the level of apoptosis produced by treatment with gemcitabine.	Pancreatic cancer cell lines BxPC3, Capan1, PancTu1	Yes	Yes	[4]
ZEB1	Transcription factor	Comparing a set of 9 pancreatic cancer cell lines to distinguish which cells are most sensitive to gemcitabine, the authors find ZEB1 expression inversely correlates with the level of apoptosis induced by gemcitabine.<br><br>Knocking down ZEB1 via siRNA increases the levels of gemcitabine-induced apoptosis.	Pancreatic cell lines SU8686, CFPAC1, L3.6P1, BxPC3, Aspc1, Mpanc96, PANC1, Miapaca2, HS766T<br><br>Pancreatic cancer cell lines PANC1, HS766T, Miapaca2	Yes	Yes	[5]
NFATC2	Transcription factor	Knocking down NFATC2 via siRNA + treatment	Pancreatic cancer cell	Yes	Yes	[6]

		with gemcitabine produces more apoptosis than siRNA control + treatment with gemcitabine.<br><br>Overexpression of NFATC2 + treatment with gemcitabine produces less apoptosis than siRNA control+ treatment with gemcitabine.	line PaTu-8988t			
SHC1	Adaptor protein	Knocking down SHC1 via siRNA + treatment with gemcitabine produces less apoptosis thank siRNA control + treatment with gemcitabine.	Pancreatic cancer cell lines PANC1, AsPC1	Yes	Yes	[7]
MUC1	membrane mucin glycoprotein	Ectopic expression of MUC1 increases resistance to gemcitabine in a pancreatic cancer cell line	Pancreatic cancer cell line BxPC3	Yes	Yes	[8]
ELK1	Transcription factor	Inactivation of ELK1 via silodosin decreased gemcitabine cytotoxicity<br><br>ELK1 is involved in transcriptional regulation and chromatin remodeling at the MYC promoter, resulting in activation of the MYC oncogene.	Prostate cancer cells<br><br>Pancreatic cancer cell line PANC1	Yes<br><br>No	No, other cancer<br><br>Yes	[9]<br><br>[10]
NKX2-1 (TTF-1)	Transcription factor	Higher expression of NKX2-1 increases gemcitabine cytotoxicity.	Lung cancer cell line NCI-H1299	Yes	No, other cancer	[11]
TGIF-1	Transcription factor	TGIF-1 overexpression leads to decreased cytotoxicity of gemcitabine.<br><br>Trichostatin A treatment reduces TGIF-1, and synergizes with gemcitabine.	Bladder cancer cell line NTUB1<br><br>NTUB1 cells continuously treated with gemcitabine such that they develop resistance	Yes	No, other cancer	[12]
PTPN1	Protein	PTPN1 expression	Breast	Correlative	No, other	[13]

(PTP-1B)	tyrosine phosphatase	correlates with gemcitabine resistance.	cancer cell lines		cancer	
STAT5A	Transcription factor	Comparing a set of 15 pancreatic cancer cell lines to distinguish which cells are most sensitive to gemcitabine, the authors find TRAF6 is relatively overexpressed in gemcitabine resistant cells.	Pancreatic cancer cell lines A818.4, AsPc-1, CFPAC-1, FA6, Hs766T, MDAPanc-3, MiaPaCa-2, PANC-1, PaTu-I, RWP-1, Suit-2, PK1, PK9, and PK59 T3M4	Correlative	Yes	[14]
TRAF6	Signaling	Comparing a set of 15 pancreatic cancer cell lines to distinguish which cells are most sensitive to gemcitabine, the authors find TRAF6 is relatively overexpressed in gemcitabine resistant cells.	Pancreatic cancer cell lines A818.4, AsPc-1, CFPAC-1, FA6, Hs766T, MDAPanc-3, MiaPaCa-2, PANC-1, PaTu-I, RWP-1, Suit-2, PK1, PK9, and PK59 T3M4	Correlative	Yes	[14]
ELK3	Transcription factor	Overexpressing ELK3 reduces cell growth in pancreatic cancer cells.	Pancreatic cell line BxPC3	Not to our knowledge	Yes	[15]
NCK1	Adaptor protein	NCK1 is necessary for cell migration induced through EGFR.	Pancreatic cancer cell line FG	Not to our knowledge	Yes	[16]
MXD1	Transcription factor	Upregulation of MXD1 via repression of miR-202 (that would otherwise repress MXD1) increases apoptosis in pancreatic cancer cells.<br><br>This increased expression of MXD1 increased its binding to SIN3A, resulting in apoptosis.	Pancreatic cancer cell line PANC1	Not to our knowledge	Yes	[17]
CRK	Adaptor protein	In patient samples, CRK is expressed at higher levels in more advanced	Lung cancer, breast cancer,	Not to our knowledge	No, other cancers	[18]

		stages of cancer.	glioblastoma			
YWHAH	Tyrosine 3-Monooxygenase/Tryptophan 5-Monooxygenase Activation Protein	YWHAH interacts with gremlin1.<br><br>Gremlin 1 is overexpressed in pancreatic cancer.	Human embryonic kidney cell line HEK293<br><br>Patient samples	Not to our knowledge	Yes	[19]
ZNF350	Transcription factor	A SNP in ZNF350 is associated with breast cancer in Caucasian populations.	Genetic association study in patients with breast and ovarian cancer vs controls	Not to our knowledge	No, other cancers	[20]
TNFRSF 11A (RANK)	TNF receptor	RANK overexpression activates NFKB.<br><br>Review of RANK roles in cancer and metastasis	Human dendritic cells<br><br>Multiple cancers	Not to our knowledge<br><br>Not to our knowledge	No cancer context in this paper<br><br>No, other cancers	[21]<br><br>[22]
ZBTB7A	Transcription factor	Many roles in different cancers:<br><br>Inactivating mutations in the zinc finger domain of ZBTB7A increase cellular proliferation.<br><br>Knockdown of ZBTB7A via siRNA in gastric cancer prevents cell migration and invasion phenotypes	 <br>Colon cancer cells SW48<br><br>Gastric cancer cells	Not to our knowledge	No, other cancers	 <br>[23]<br><br>[24]
IRF7	Transcription factor	Overexpression of IRF7 reduces metastasis	Mouse model with mammary gland tumor that metastasizes to the bone	Not to our knowledge	No, other cancer	[25]
TLX2	Transcription factor	No conclusive studies about TLX2 in cancer				
PRKCZ	Kinase	In S2-CP9 cells, inhibition of PRKCZ reduces cell adhesion, preventing cell migration.	Pancreatic cancer cell line SUIT-2 clones S2-m (little metasis) and S2-CP9 (highly	Not to our knowledge	Yes	[26]

			metastatic)			

References:

[1] J. Hou and L. Wang, “FKBP5 as a selection biomarker for gemcitabine and Akt inhibitors in treatment of pancreatic cancer.,” *PLoS One*, vol. 7, no. 5, p. e36252, Jan. 2012.

[2] W.-T. Wei, H. Chen, Z.-H. Wang, Z.-L. Ni, H.-B. Liu, H.-F. Tong, H.-C. Guo, D.-L. Liu, and S.-Z. Lin, “Enhanced antitumor efficacy of gemcitabine by evodiamine on pancreatic cancer via regulating PI3K/Akt pathway.,” *Int. J. Biol. Sci.*, vol. 8, no. 1, pp. 1–14, Jan. 2012.

[3] H. L. Kindler, M. Aklilu, S. Nattam, and E. E. Vokes, “Arsenic trioxide in patients with adenocarcinoma of the pancreas refractory to gemcitabine: a phase II trial of the University of Chicago Phase II Consortium.,” *Am. J. Clin. Oncol.*, vol. 31, no. 6, pp. 553–6, Dec. 2008.

[4] A. Arlt, A. Gehrz, S. Müerköster, J. Vorndamm, M.-L. Kruse, U. R. Fölsch, and H. Schäfer, “Role of NF-kappaB and Akt/PI3K in the resistance of pancreatic carcinoma cell lines against gemcitabine-induced cell death.,” *Oncogene*, vol. 22, no. 21, pp. 3243–51, May 2003.

[5] T. Arumugam, V. Ramachandran, K. F. Fournier, H. Wang, L. Marquis, J. L. Abbruzzese, G. E. Gallick, C. D. Logsdon, D. J. McConkey, and W. Choi, “Epithelial to mesenchymal transition contributes to drug resistance in pancreatic cancer.,” *Cancer Res.*, vol. 69, no. 14, pp. 5820–8, Jul. 2009.

[6] H. Griesmann, S. Ripka, M. Pralle, V. Ellenrieder, S. Baumgart, M. Buchholz, C. Pilarsky, D. Aust, T. M. Gress, and P. Michl, “WNT5A-NFAT signaling mediates resistance to apoptosis in pancreatic cancer.,” *Neoplasia*, vol. 15, no. 1, pp. 11–22, Jan. 2013.

[7] S. Hamada, A. Masamune, S. Miura, K. Satoh, and T. Shimosegawa, “MiR-365 induces gemcitabine resistance in pancreatic cancer cells by targeting the adaptor protein SHC1 and pro-apoptotic regulator BAX.,” *Cell. Signal.*, vol. 26, no. 2, pp. 179–85, Feb. 2014.

[8] S. Nath, K. Daneshvar, L. D. Roy, P. Grover, A. Kidiyoor, L. Mosley, M. Sahraei, and P. Mukherjee, “MUC1 induces drug resistance in pancreatic cancer cells via upregulation of multidrug resistance genes.,” *Oncogenesis*, vol. 2, p. e51, Jan. 2013.

[9] T. Kawahara, A. K. Aljarah, H. K. Shareef, S. Inoue, H. Ide, J. D. Patterson, E. Kashiwagi, B. Han, Y. Li, Y. Zheng, and H. Miyamoto, “Silodosin inhibits prostate cancer cell growth via ELK1 inactivation and enhances the cytotoxic activity of gemcitabine.,” *Prostate*, Feb. 2016.

[10] A. Köenig, T. Linhart, K. Schlengemann, K. Reutlinger, J. Wegele, G. Adler, G. Singh, L. Hofmann, S. Kunsch, T. Büch, E. Schäfer, T. M. Gress, M. E. Fernandez-Zapico, and V. Ellenrieder, “NFAT-induced histone acetylation relay switch promotes c-Myc-dependent growth in pancreatic cancer cells.,” *Gastroenterology*, vol. 138, no. 3, pp. 1189-99–2, Mar. 2010.

[11] L. Yang, M. Lin, W. Ruan, L. Dong, E. Chen, X. Wu, and K. Ying, “Nkx2-1: a novel tumor biomarker of lung cancer.,” *J. Zhejiang Univ. Sci. B*, vol. 13, no. 11, pp. 855–66, Nov. 2012.

[12] B.-W. Yeh, W.-M. Li, C.-C. Li, W.-Y. Kang, C.-N. Huang, T.-C. Hour, Z.-M. Liu, W.-J. Wu, and H.-S. Huang, “Histone deacetylase inhibitor trichostatin A resensitizes gemcitabine resistant urothelial carcinoma cells via suppression of TG-interacting factor.,” *Toxicol. Appl. Pharmacol.*, vol. 290, pp. 98–106, Jan. 2016.
[13] K. Shen, S. D. Rice, D. A. Gingrich, D. Wang, Z. Mi, C. Tian, Z. Ding, S. L. Brower, P. R. Ervin, M. J. Gabrin, G. Tseng, and N. Song, “Distinct genes related to drug response identified in ER positive and ER negative breast cancer cell lines.,” *PLoS One*, vol. 7, no. 7, p. e40900, Jan. 2012.
[14] M. Akada, T. Crnogorac-Jurcevic, S. Lattimore, P. Mahon, R. Lopes, M. Sunamura, S. Matsuno, and N. R. Lemoine, “Intrinsic chemoresistance to gemcitabine is associated with decreased expression of BNIP3 in pancreatic cancer.,” *Clin. Cancer Res.*, vol. 11, no. 8, pp. 3094–101, Apr. 2005.
[15] B. Li, P. Ni, Q. Zhu, H. Cao, H. Xu, S. Zhang, C. Au, and Y. Zhang, “Growth inhibitory effect of the ternary complex factor Net on human pancreatic carcinoma cell lines.,” *Tohoku J. Exp. Med.*, vol. 216, no. 2, pp. 139–47, Oct. 2008.
[16] M. Huang, S. Anand, E. A. Murphy, J. S. Desgrosellier, D. G. Stupack, and S. J. Shattil, “EGFR-dependent pancreatic carcinoma cell metastasis through Rap1 activation,” vol. 31, no. 22, pp. 2783–2793, 2011.
[17] L. Farhana, M. I. Dawson, and J. A. Fontana, “Down regulation of miR-202 modulates Mxd1 and Sin3A repressor complexes to induce apoptosis of pancreatic cancer cells.,” *Cancer Biol. Ther.*, vol. 16, no. 1, pp. 115–24, Jan. 2015.
[18] S. Kumar, J. E. Fajardo, R. B. Birge, and G. Sriram, “Crk at the quarter century mark: perspectives in signaling and cancer.,” *J. Cell. Biochem.*, vol. 115, no. 5, pp. 819–25, May 2014.
[19] H. Namkoong, S. M. Shin, H. K. Kim, S.-A. Ha, G. W. Cho, S. Y. Hur, T. E. Kim, and J. W. Kim, “The bone morphogenetic protein antagonist gremlin 1 is overexpressed in human cancers and interacts with YWHAH protein.,” *BMC Cancer*, vol. 6, no. 1, p. 74, Jan. 2006.
[20] M. E. Sehl, L. R. Langer, J. C. Papp, L. Kwan, J. L. Seldon, G. Arellano, J. Reiss, E. F. Reed, S. Dandekar, Y. Korin, J. S. Sinsheimer, Z.-F. Zhang, and P. A. Ganz, “Associations between single nucleotide polymorphisms in double-stranded DNA repair pathway genes and familial breast cancer.,” *Clin. Cancer Res.*, vol. 15, no. 6, pp. 2192–203, Mar. 2009.
[21] D. M. Anderson, E. Maraskovsky, W. L. Billingsley, W. C. Dougall, M. E. Tometsko, E. R. Roux, M. C. Teepe, R. F. DuBose, D. Cosman, and L. Galibert, “A homologue of the TNF receptor and its ligand enhance T-cell growth and dendritic-cell function.,” *Nature*, vol. 390, no. 6656, pp. 175–179, Nov. 1997.
[22] E. González-Suárez and A. Sanz-Moreno, “RANK as a therapeutic target in cancer,” *FEBS J.*, vol. 283, no. 11, pp. 2018–2033, Jun. 2016.
[23] X.-S. Liu, Z. Liu, C. Gerarduzzi, D. E. Choi, S. Ganapathy, P. P. Pandolfi, and Z.-M. Yuan, “Somatic human ZBTB7A zinc finger mutations promote cancer progression.,” *Oncogene*, Oct. 2015.
[24] D.-B. Shi, Y.-W. Wang, A.-Y. Xing, J.-W. Gao, H. Zhang, X.-Y. Guo, and P. Gao, “C/EBPα-induced miR-100 expression suppresses tumor metastasis and

growth by targeting ZBTB7A in gastric cancer.," *Cancer Lett.*, vol. 369, no. 2, pp. 376–85, Dec. 2015.

[25] B. N. Bidwell, C. Y. Slaney, N. P. Withana, S. Forster, Y. Cao, S. Loi, D. Andrews, T. Mikeska, N. E. Mangan, S. A. Samarajiwa, N. A. de Weerd, J. Gould, P. Argani, A. Möller, M. J. Smyth, R. L. Anderson, P. J. Hertzog, and B. S. Parker, "Silencing of Irf7 pathways in breast cancer cells promotes bone metastasis through immune escape," *Nat. Med.*, vol. 18, no. 8, pp. 1224–1231, Jul. 2012.

[26] M. Della Peruta, C. Giagulli, C. Laudanna, A. Scarpa, and C. Sorio, "RHOA and PRKCZ control different aspects of cell motility in pancreatic cancer metastatic clones.," *Mol. Cancer*, vol. 9, no. 1, p. 61, Jan. 2010.
